# Regional Blocks in the Era of the Opioid Crisis: Evaluating Their Opioid-Sparing Effect

**DOI:** 10.7759/cureus.109997

**Published:** 2026-05-31

**Authors:** Sneha Ann Johnson, Dilip Kumar G, Jyothi Susan Thomas, Vishnu Sadanandan, Sreenivas Umaiorubahan Meenakshisundaram, Sony Prakkattumannathu Varghese, Anant C Fulse

**Affiliations:** 1 Anesthesiology, Shri Satya Sai Medical College and Research Institute, Chennai, IND; 2 Obstetrics and Gynecology, Badr Al Samaa Hospital, Ruwi, OMN; 3 Neurology, Apollo Speciality Hospital, Vanagaram, Chennai, IND; 4 Plastic Surgery, Khoula Hospital, Muscat, OMN; 5 Anatomy, NKP Salve Institute of Medical Sciences and Research Centre and Lata Mangeshkar Hospital, Nagpur, IND

**Keywords:** multimodal analgesia, opioid crisis, opioid-sparing analgesia, perioperative pain management, peripheral nerve blocks, regional anesthesia

## Abstract

Perioperative pain management strategies are increasingly shifting towards opioid-sparing approaches due to the ongoing opioid epidemic, with regional anaesthesia contributing significantly to multimodal analgesia. The purpose of this narrative review was to determine if the effect of regional anaesthesia on opioid consumption has been overestimated. Literature searches performed through PubMed, MEDLINE, and Google Scholar identified relevant studies that evaluated perioperative consumption of opioids, postoperative pain, and recovery profiles for neuraxial and peripheral nerve blocks. Current evidence demonstrates that regional anaesthesia significantly reduces opioid requirements and improves pain control in the immediate postoperative period, typically within the first 24-48 hours, while also decreasing opioid-related adverse effects such as postoperative nausea, sedation, and respiratory depression. However, most studies are limited to short-term outcomes, with insufficient evidence supporting sustained reductions in long-term opioid use or prevention of chronic pain. Furthermore, substantial heterogeneity in study design, surgical procedures, and concurrent use of multimodal analgesia complicates the isolation of regional anaesthesia's independent effect, potentially leading to overestimation of its true impact. Additional factors, including rebound pain, limited duration of single-shot blocks, and practical implementation constraints, may further attenuate its clinical benefit. While regional anaesthesia remains an important component of perioperative analgesia, its opioid-sparing effect appears to be context-dependent and best understood as part of a balanced, individualised multimodal approach rather than as a standalone solution.

## Introduction and background

The increased incidence of opioid abuse, dependence, and overdose has led many organizations to change how they manage pain in the perioperative period to include more opioid-sparing techniques. Even though opioids are an effective way to relieve pain, their use is limited by side effects such as respiratory depression, nausea, vomiting, sedation, and opioid-induced hyperalgesia [1]. Opioid-sparing strategies have also been explored beyond the perioperative setting, particularly in critically ill patients and in chronic pain management, where minimizing opioid exposure has been associated with improved safety profiles and reduced risk of long-term dependence. In intensive care settings, multimodal analgesia and opioid minimization approaches have been linked to decreased duration of mechanical ventilation and improved recovery outcomes. These broader applications highlight the evolving emphasis on reducing opioid reliance across diverse clinical contexts [2,3].

As such, multimodal approaches to pain relief are being used more frequently, especially as part of Enhanced Recovery After Surgery (ERAS) programs, to help manage pain with less reliance on opioids. In conjunction with this shift away from routine opioid use, regional anesthesia techniques-broadly categorized into neuraxial blocks (spinal and epidural anesthesia) and peripheral nerve blocks-are increasingly utilized to provide targeted analgesia and reduce systemic opioid requirements [4]. In this context, the term opioid-sparing effect refers primarily to a reduction in perioperative opioid consumption, particularly during the immediate postoperative period, as well as its potential influence on longer-term opioid use, functional recovery, and patient-centered outcomes. However, despite widespread adoption of these techniques, there remains uncertainty regarding the magnitude and clinical significance of their opioid-sparing benefits in the perioperative setting compared to current perceptions in clinical practice [1,4,5].

## Review

A narrative review was conducted to evaluate the opioid-sparing effect of regional anesthesia in the perioperative setting. A structured literature search was performed using PubMed, MEDLINE, and Google Scholar to identify relevant studies published between 2010 and 2026. Search terms included “regional anesthesia,” “opioid-sparing,” “multimodal analgesia,” “peripheral nerve blocks,” and “opioid crisis.” Eligible studies included randomized controlled trials, observational studies, systematic reviews, and meta-analyses that evaluated perioperative opioid consumption, postoperative pain outcomes, and recovery profiles. Studies were selected based on clinical relevance, methodological quality, and applicability to perioperative practice. Preference was given to higher-level evidence, including systematic reviews and randomized controlled trials.

The review focused on both short-term outcomes (e.g., opioid consumption within 24-48 hours and early postoperative pain scores) and long-term outcomes (e.g., persistent opioid use and functional recovery). Particular attention was given to heterogeneity across surgical procedures and techniques, as well as confounding factors such as the concurrent use of multimodal analgesia. As this is a narrative review, formal systematic review protocols such as the PRISMA guidelines were not applied; however, efforts were made to ensure transparency, comprehensive literature coverage, and a balanced interpretation of the available evidence [6].

Rationale for opioid-sparing strategies

There is increasing concern regarding the adverse effects associated with long-term and perioperative opioid use, which has driven a shift toward opioid-sparing strategies in modern perioperative care. Opioid administration is associated with numerous side effects, including postoperative nausea and vomiting (PONV), respiratory depression, oversedation, and delayed recovery, all of which can negatively impact patient outcomes and prolong hospital stay [1,4,5]. In particular, opioids are a significant contributor to PONV, which can impair patient comfort, delay oral intake, and extend hospitalization, while the use of regional anesthesia and multimodal analgesia may reduce opioid consumption and thereby decrease the incidence of PONV, improving early postoperative recovery [7,8]. Respiratory depression remains one of the most serious opioid-related complications, especially in high-risk populations such as the elderly and patients with comorbidities, highlighting the importance of opioid-sparing strategies, including regional anesthesia, in mitigating this potentially life-threatening risk [9]. Additionally, opioid-induced sedation can impair cognitive function, delay mobilization, and prolong recovery from anesthesia, whereas reducing opioid exposure may facilitate faster awakening, earlier ambulation, and improved recovery trajectories in line with perioperative care goals [10]. Furthermore, perioperative opioid use has been identified as a risk factor for persistent postoperative opioid use, even in previously opioid-naïve patients, thereby contributing to the ongoing opioid epidemic; consequently, multimodal analgesia approaches incorporating regional blocks and non-opioid medications are increasingly utilized to limit opioid exposure and potentially reduce the risk of long-term dependence, although the precise magnitude of opioid reduction achieved by each modality remains under investigation [1,11].

Regional anesthesia: mechanisms of opioid sparing

Acting on the origin of pain pathways with regional anaesthesia lessens the need for systemic analgesics through an opioid-sparing effect. Regional techniques effectively reduce the amount of afferent pain signalling to the central nervous system by interrupting nociceptive transmission either peripherally or neuraxially. By providing superior site-specific analgesia and interrupting the mechanisms of central sensitisation, key contributors to amplified postoperative pain and hyperalgesia, regional anaesthesia enhances postoperative pain control and reduces the incidence of systemic side effects compared to opioid-based regimens alone. In addition to reducing peripheral nociceptive input, regional anesthesia plays a key role in limiting central sensitization, a process in which repeated or sustained noxious stimuli amplify pain perception and contribute to postoperative hyperalgesia [12]. By attenuating this process, regional techniques help prevent the escalation of pain intensity and reduce subsequent opioid requirements. Neuraxial blocks (e.g., spinal or epidural anaesthesia) inhibit pain transmission at the spinal cord level, while peripheral nerve blocks (e.g., transversus abdominis plane (TAP), paravertebral, femoral, erector spinae) target specific peripheral nerves that provide sensory innervation to the surgical site. As a result of these regional anaesthetic techniques, intraoperative and early postoperative systemic opioid requirement is reduced significantly. Despite the well-known physiological basis for the opioid-sparing effects of regional anaesthesia, the degree to which the effects of these mechanisms lead to long-term clinical benefits continues to be researched, particularly when regional anaesthesia is used in combination with the other elements of a multimodal analgesia regimen [1,13,14]. However, while these mechanisms provide a strong physiological basis for opioid reduction, their translation into consistent long-term clinical benefits remains uncertain, particularly when regional anesthesia is used alongside other components of multimodal analgesia.

Table 1 provides a summary of the main groups of regional anesthesia techniques, together with their indicated clinical uses and relative magnitude of opioid-sparing effects. It can be seen that while early opioid sparing with neuraxial blocks is substantial, the efficacy of peripheral and fascial plane blocks is dependent upon both surgical context, technique, and duration of analgesia [15].

**Table 1:** Types of regional blocks and their opioid-sparing potential TAP: transversus abdominis plane.

Block Type	Examples	Common Surgeries	Opioid-Sparing Effect
Neuraxial	Spinal, Epidural	Abdominal, Obstetric	High (early phase)
Peripheral	TAP, Femoral, Paravertebral	Orthopedic, Thoracic	Moderate–High
Fascial Plane	Erector Spinae, Quadratus Lumborum	Abdominal, Spine	Variable

Evidence supporting opioid-sparing effects

A substantial body of evidence from randomized controlled trials and systematic reviews demonstrates that regional anesthesia techniques reduce perioperative opioid requirements, particularly in the immediate postoperative period. Numerous clinical studies and systematic reviews have shown extremely strong evidence for the use of regional anesthesia (especially peripheral nerve blocks) to decrease opioid use, with many studies reporting reductions in opioid requirements, typically ranging from 30-50% depending on the surgical context and regional technique used (even in children with musculoskeletal injuries) [16]. Regional anesthesia can produce lower postoperative opioid requirements; however, the research strongly demonstrates that the use of regional anesthesia will also produce better early postoperative outcomes. Patients who receive regional anesthesia techniques report lower levels of pain throughout the first 24-48 hours following surgery, have less PONV, and can mobilize and recover functionally sooner after surgery than patients who received general anaesthetic alone [17]. The decreased opioid requirements and improved early postoperative outcomes associated with the use of regional anesthesia also coincide with many of the objectives of ERAS protocols [18]. While the above results support the incorporation of regional anesthesia techniques into multimodal analgesic plans, the results should only be interpreted as being limited to their immediate postoperative benefits, and therefore, it is not possible to determine how patients will use opioids or recover from surgery after the initial postoperative period following surgery due to the lack of available data [18,19]. The magnitude of opioid reduction varies depending on surgical procedure, type of regional block, and comparator analgesic strategy. Greater reductions are consistently observed in orthopedic and thoracic procedures, where targeted nerve blockade directly addresses surgical pain pathways. In contrast, benefits may be less pronounced in procedures with diffuse or visceral pain components. Additionally, most studies report outcomes within the first 24-48 hours following surgery, with limited evidence supporting sustained long-term reductions in opioid use [20].

Limitations of current evidence

**Figure 1:**
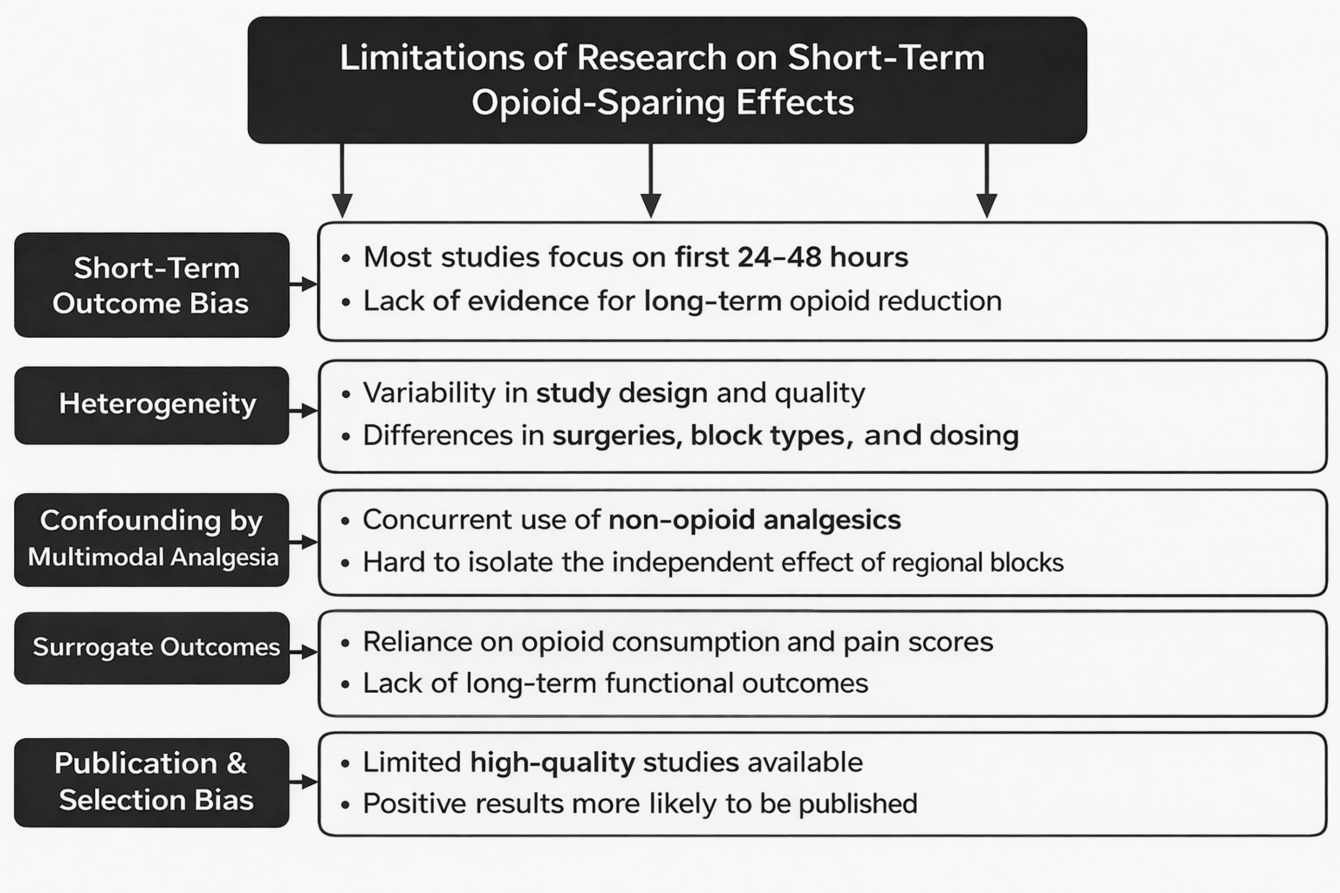
Limitations of current evidence

Regional anaesthesia has demonstrated benefits in reducing early postoperative opioid consumption; however, several methodological and clinical limitations complicate the interpretation of its true opioid-sparing effect [21]. These limitations are summarized in Figure 1.

Short-Term Outcomes and Clinical Relevance

Many studies evaluating regional anaesthesia rely predominantly on short-term outcomes, such as opioid consumption within the first 24-48 hours and early postoperative pain scores. While these studies consistently demonstrate reductions in opioid use and improved early analgesia, there is limited evidence regarding long-term outcomes, including sustained opioid reduction, prevention of chronic postoperative pain, and functional recovery [22]. Furthermore, reduced opioid consumption does not always correlate with meaningful improvements in patient-centered outcomes such as satisfaction, complication rates, or overall recovery, raising concerns regarding the clinical relevance of commonly used surrogate endpoints. In addition, publication bias and enthusiasm bias may favor studies reporting positive findings, potentially leading to an overestimation of the true clinical impact of regional anaesthesia [23]. Additionally, publication bias may favor studies reporting positive outcomes, potentially leading to an overrepresentation of beneficial effects and contributing to an overestimation of the opioid-sparing impact of regional anesthesia.

Heterogeneity and Study Design Limitations

A major limitation of the current literature is the significant heterogeneity across studies evaluating regional anaesthesia. Variations in surgical procedures, block techniques, local anaesthetic agents, and outcome measures make direct comparisons challenging and limit generalizability [24]. Additionally, differences in study design and institutional practices further complicate the interpretation of results, making it difficult to accurately quantify the independent opioid-sparing effect of regional techniques [25]. This variability limits the generalizability of findings and complicates direct comparison across studies.

Confounding by Multimodal Analgesia and Clinical Context

The use of regional anesthesia as part of multimodal analgesia limits the ability to accurately assess its independent effect on opioid reduction. Regional techniques are frequently combined with multiple non-opioid analgesic agents, including nonsteroidal anti-inflammatory drugs (NSAIDs), paracetamol, N-methyl-D-aspartate (NMDA) receptor antagonists such as ketamine, and alpha-2 agonists such as clonidine or dexmedetomidine, all of which contribute to analgesia through complementary mechanisms, including anti-inflammatory effects, reduction in central sensitization, and synergistic analgesia. NSAIDs reduce inflammation and peripheral sensitization [26], paracetamol provides central baseline analgesia, NMDA receptor antagonists prevent central sensitization through receptor blockade [27], and alpha-2 agonists enhance both analgesia and sedation while potentiating regional techniques [28]. The concurrent use of these agents creates methodological challenges in isolating and quantifying the specific contribution of regional anesthesia to opioid reduction, and many clinical studies report decreased opioid use following regional anesthesia without adequately controlling for these adjuncts [29]. This overlap makes it difficult to isolate the independent contribution of regional anesthesia, as reductions in opioid use may reflect the cumulative effect of multimodal strategies rather than a single intervention. Additionally, variability in multimodal protocols across institutions further complicates comparison of results and interpretation of findings [30], while differences in study design and implementation introduce further complexity [31].

Furthermore, although regional anesthesia has consistently been associated with reduced opioid consumption, this reduction does not always translate into meaningful improvements in patient-centered outcomes such as satisfaction, complication rates, or functional recovery. Some studies demonstrate only modest changes in pain scores despite decreased opioid use, raising concerns about the clinical relevance of opioid consumption as a surrogate endpoint [32]. Evidence from clinical studies suggests that regional techniques can reduce opioid requirements and improve early postoperative analgesia [1], with systematic reviews reporting reductions of approximately 30-50% [16], particularly in orthopedic, thoracic, and abdominal surgeries [33]. However, these benefits are largely confined to the immediate postoperative period, and data on long-term outcomes remain limited. Moreover, increasing adoption of multimodal analgesia regimens, combined with variability in block techniques and rebound pain following single-shot blocks, further complicates interpretation of results [34], while heterogeneity in study design and clinical practice limits generalizability [35]. The lack of consistent correlation between opioid reduction and functional recovery further challenges the interpretation of these findings [36]. As a result, the independent benefit of regional anesthesia remains difficult to quantify, underscoring the need for cautious, context-based evaluation of its role within perioperative pain management.

Overestimating the opioid-sparing effect

Magnitude and Clinical Significance of Effect

Although numerous studies demonstrate statistically significant reductions in opioid consumption with regional anaesthetic techniques, the overall clinical magnitude of these reductions is often modest when compared to other analgesic strategies, including systemic opioids. The observed benefits are largely confined to the immediate postoperative period, particularly within the first 24-48 hours, with limited evidence supporting sustained long-term outcomes such as reduced chronic opioid use or improved functional recovery [36]. This discrepancy raises concerns regarding the clinical significance of opioid reduction as a standalone outcome and suggests that its impact may be overstated when not evaluated in a broader clinical context [37].

Context-Dependent Efficacy and Technique Limitations

The degree to which regional anaesthesia produces satisfactory outcomes is influenced by many independent and interdependent contextual factors, such as surgical procedure type, accuracy of technical performance, and patient-related characteristics or variables. In general, orthopedic and thoracic procedures produce more substantial advantages than other surgical procedures. Additionally, there is a marked variability in the quality, duration, and method of administering blocks, as well as in the characteristics of patients, including their baseline levels of pain sensitivity, psychological status, and the degree of previous exposure to opioid medications. Such variability can have a significant impact on the outcome for each individual [38,39]. There is also the potential for rebound pain, evidenced by an increase in the intensity of pain after the regional anaesthesia block has been resolved, associated with the transient nature of the anesthetic used in a single-shot regional anaesthesia technique. This may, in turn, lead to an increase in opioid use during the late postoperative period and diminish the overall ability of the regional anaesthesia to serve as a long-term opioid-sparing modality [8].

Practical and Implementation Constraints

In real-world clinical practice, several logistical and practical challenges limit the widespread and consistent application of regional anaesthesia. These include the requirement for specialized training, availability of ultrasound guidance, time constraints in busy operating room settings, and the potential for complications such as nerve injury or local anaesthetic systemic toxicity. Such factors may restrict the routine use of regional techniques and influence their effectiveness across different clinical environments [1,18].

Role of regional anesthesia within multimodal analgesia

Regional Anesthesia as a Component of Multimodal Analgesia

In the context of modern perioperative pain management, regional anaesthesia should be viewed as an integral component of a multimodal analgesia strategy rather than a standalone intervention [34,40]. Multimodal approaches utilize a combination of pharmacological and non-pharmacological techniques targeting different pain pathways to optimize analgesia while minimizing opioid-related adverse effects [41]. Regional blocks contribute significantly to early postoperative pain control and reduction in opioid requirements; however, their effects are complementary to other modalities such as nonsteroidal anti-inflammatory drugs (NSAIDs), paracetamol, N-methyl-D-aspartate (NMDA) receptor antagonists, and adjuvant agents [42,43]. Importantly, the goal of perioperative analgesia is not the complete elimination of opioids but rather their judicious and individualized use based on patient needs, surgical factors, and response to therapy [44]. While regional techniques can reduce opioid consumption, they do not consistently demonstrate superiority over non-opioid-based or traditional analgesic strategies across all surgical populations, reinforcing the need for individualized analgesic planning [45,46]. Regional anesthesia should therefore be viewed as a complementary component within a broader analgesic strategy rather than an independent substitute for opioids.

Evidence, Limitations, and Contextual Interpretation

This growing body of literature supports the benefit of regional anaesthesia via multimodal analgesia with respect to a reduction in opioid consumption and improvement in early postoperative pain outcomes. Several studies, including those by Kehlet et al. and Memtsoudis et al., have shown that the addition of regional anaesthesia to multimodal analgesia improves perioperative recovery and results in less requirement for opioids, particularly in the case of orthopedic and thoracic procedures [46,47]. These benefits are most consistently observed in the immediate postoperative period and are often influenced by the type of surgery and the accompanying multimodal analgesic regimen. However, the majority of these findings are seen only during the early postoperative period, and the data are limited, showing no evidence of preventing long-term dependence upon opioids via the use of regional anaesthesia. For example, Sun et al. demonstrated an association between perioperative opioid exposure and prolonged postoperative use; however, there is no evidence to support that regional anaesthesia alone prevents long-term dependence upon opioids [48]. This highlights that while regional anesthesia may reduce initial opioid exposure, it does not independently prevent long-term opioid dependence.

The interpretation of the findings is confounded by several factors. Multimodal analgesia contributes to the control of pain significantly, making it difficult to differentiate the independent effect of regional anaesthesia [38,39]. Several other challenges also limit the generalizability of these results, including rebound pain from the use of a single-shot block; variability in both the clinical practice and technique for administering a block; and heterogeneity in the design of the studies [48,49]. In addition, the use of surrogate endpoints (e.g., opioid consumption) may not necessarily reflect clinically meaningful outcomes because there is no correlation between reductions in the use of opioids and increased patient satisfaction, recovery of function, and quality of overall recovery [50,51]. The concurrent use of multiple analgesic modalities makes it inherently difficult to isolate the independent contribution of regional anesthesia to opioid reduction.

Clinical Implications and Balanced Approach

Taken together, these findings suggest that while regional anaesthesia plays a valuable role in perioperative pain management, its benefits are context-dependent and should be interpreted within the broader framework of multimodal analgesia. A balanced approach that combines regional techniques with other analgesic modalities, while allowing for appropriate opioid use when necessary, is essential to achieve optimal patient outcomes. Complete opioid elimination is neither feasible nor appropriate for all patients, given variability in pain perception, surgical complexity, and individual response to analgesia. Therefore, regional anaesthesia should be incorporated as part of an individualized, patient-centered analgesic plan rather than being considered a universal substitute for opioids.

Overall, current evidence supports the role of regional anaesthesia in improving early postoperative outcomes and reducing opioid consumption; however, its long-term impact remains uncertain, emphasizing the need for cautious interpretation and continued research. Key studies evaluating the opioid-sparing role of regional anesthesia and multimodal analgesia are summarized in Table 2.

**Table 2:** Summary of key studies evaluating the opioid-sparing effect of regional anesthesia and multimodal analgesia

Study	Study Type	Clinical Setting	Main Findings	Key Limitation
Kehlet et al. [46]	Narrative review	Postoperative pain management	Multimodal analgesia with regional techniques reduced opioid requirements and improved recovery	Primarily focused on short-term outcomes
Opperer et al. [47]	Review	Orthopedic and thoracic surgery	Regional anesthesia improved perioperative outcomes and reduced postoperative opioid use	Procedure-specific applicability
Sun et al. [48]	Cohort study	Opioid-naïve postoperative patients	Perioperative opioid exposure was associated with prolonged postoperative opioid use	Did not establish the independent preventive role of regional anesthesia
Lavand’homme [49]	Review	Ambulatory surgery	Rebound pain after single-shot blocks may increase postoperative opioid requirements	Limited long-term outcome assessment
Joshi et al. [50]	Review	Multiple surgical procedures	Variability in surgical procedures and analgesic protocols affects the interpretation of opioid-sparing outcomes	Significant heterogeneity across studies
Zorrilla-Vaca et al. [51]	Cohort study	Open gynecologic surgery	Reduced opioid consumption did not consistently correlate with improved patient-reported outcomes	Surrogate endpoint limitations
Soffin et al. [35]	Narrative review	Total joint arthroplasty	Regional and multimodal analgesia reduced opioid use and improved early recovery	Limited evidence for long-term opioid reduction
Zhang et al. [18]	Systematic review and meta-analysis	Postoperative patients	Opioid-sparing strategies improved postoperative pain control and recovery profiles	Variability in included analgesic protocols
Winoker et al. [16]	Systematic review	Percutaneous nephrolithotomy	Peripheral nerve blocks reduced postoperative opioid requirements	Limited generalizability outside urologic surgery
Chou et al. [39]	Clinical practice guideline	Postoperative pain management	Multimodal analgesia is recommended to reduce opioid exposure and improve analgesia	Broad recommendations, not procedure-specific

## Conclusions

Regional analgesia continues to play a significant role in today's perioperative pain management, providing adequate pain relief as well as reducing opioid use primarily in the immediate post-operative period. There is now more evidence that the effect of regional anesthesia on opioid-sparing may depend on the context in which it is used and may be overestimated when viewed independently of the overall strategy of multimodal analgesia. Variability among surgical procedures, patient characteristics, and concomitant analgesic use can each affect the overall effect of regional anaesthetic techniques. Consequently, an individualized, balanced method using a combination of regional and other pharmacological or non-pharmacological approaches is required to ensure optimal pain relief while minimizing the likelihood of adverse events from opioids.
